# Matrix-entrapped fibers create ecological niches for gut bacterial growth

**DOI:** 10.1038/s41598-023-27907-7

**Published:** 2023-02-02

**Authors:** Nuseybe Bulut, Thaisa M. Cantu-Jungles, Xiaowei Zhang, Zeynep Mutlu, Mukerrem Cakmak, Bruce R. Hamaker

**Affiliations:** 1 Whistler Center for Carbohydrate Research and Department of Food Science, Purdue University, West Lafayette, IN USA; 2 School of Health Science and Engineering, University of Shanghai for Science and Technology, Shanghai, 200093 China; 3 School of Materials Engineering, School of Mechanical Engineering and Birck Nanotechnology Center, Purdue University, West Lafayette, IN 47907 USA

**Keywords:** Microbiology, Applied microbiology, Microbial communities

## Abstract

Insoluble plant cell walls are a main source of dietary fiber. Both chemical and physical fiber structures create distinct niches for gut bacterial utilization. Here, we have taken key fermentable solubilized polysaccharides of plant cell walls and fabricated them back into cell wall-like film forms to understand how fiber physical structure directs gut bacterial fermentation outcomes. Solubilized corn bran arabinoxylan (Cax), extracted to retain some ferulate residues, was covalently linked using laccase to form an insoluble cell wall-like film (Cax-F) that was further embedded with pectin (CaxP-F). In vitro fecal fermentation using gut microbiota from three donors was performed on the films and soluble fibers. Depending on the donor, CaxP-F led to higher relative abundance of recognized beneficial bacteria and/or butyrate producers—*Akkermansia*, *Bifidobacterium*, *Eubacterium halii*, unassigned *Lachnospiraceae*, *Blautia*, and *Anaerostipes*—than free pectin and Cax, and Cax-F. Thus, physical form and location of fibers within cell walls form niches for some health-related gut bacteria. This work brings a new understanding of the importance of insoluble cell wall-associated fibers and shows that targeted fiber materials can be fabricated to support important gut microbiota taxa and metabolites of health significance.

## Introduction

The gut microbiome is a complex community of over a trillion microbial cells which have an important role in human physiology and health^1^. Depending on its composition and balance^2^, as well as fermentation metabolites such as short chain fatty acids (SCFA; mainly acetate, butyrate and propionate), ones’ microbiota exists in a healthy state^3^. Dietary fibers are known to maintain and/or modify the colonic microbiota composition^4–6^. The composition of dietary fibers, including chemical structure (i.e. linkage type, monosaccharide composition, molecular size) and physical characteristics (solubility, insoluble three-dimensional structure) are critical factors to shape the human gut microbiota^7^.

Different gut bacterial groups have various ways to access, degrade, and utilize dietary fibers. For instance, while the gram (−) Bacteroidetes physically bind to fiber substrates through their membrane-bound SUS-like systems, many gram ( +) Firmicutes have cellulosome-like appendages that can extend into insoluble plant cell wall matrices^8^. Previous studies from our group have shown that the physical characteristics of dietary fiber can affect the gut microbial community, and understanding this relationship is one of the factors necessary to understand how to design fibers for predictable shifts in the gut microbiota^9,10^. Not only do chemical structures of fibers align to specific bacteria and bacterial groups, also physical structure targets them to specific groups. For example, we found that resistant starch entrapped in a porous alginate matrix promoted butyrogenic Firmicutes in a mouse gut microbiota, and free resistant starch promoted Bacteroidetes^9^. Hence, the chemically simple resistant starch promoted different bacteria once made physically complex.

Fibers consumed in diets mainly come from plant cell walls that are mostly in the form of matrices comprised of insoluble crosslinked polysaccharides and entrapped soluble fibers (e.g. pectin)^11^. In cereals, arabinoxylan is the main crosslinked polysaccharide consisting, in its simplest form, of a β (1,4)-linked xylan backbone substituted with arabinose residues^12^. Ferulic acid is bound to some arabinose and crosslinks the polymer chains to create, with cellulose, the cell wall matrix network^13^. Saulnier & Thibault^14^ showed that the insolubility of arabinoxylan in corn bran cell walls is due to the relatively high crosslinking through diferulate and triferulate bridges.

Arabinoxylan can be isolated from cereal cell walls by alkali treatment^15–18^, which de-esterifies the crosslinks to release soluble arabinoxylan. Because not all of the ferulate moieties on the arabinose branches are crosslinked and their lability to alkali differs, those retained on the soluble arabinoxylan can be used to create new crosslinks with oxidases like laccase^19,20^. Recently in our group, Zhang et al.^10^ used this approach to obtain soluble crosslinked matrices that promoted Firmicutes butyrogenic Clostridial bacteria. Butyrate is an important short chain fatty acid fermentation metabolite that is associated with good gut health through reduced inflammation and good barrier function^21^.

**Figure 1:**
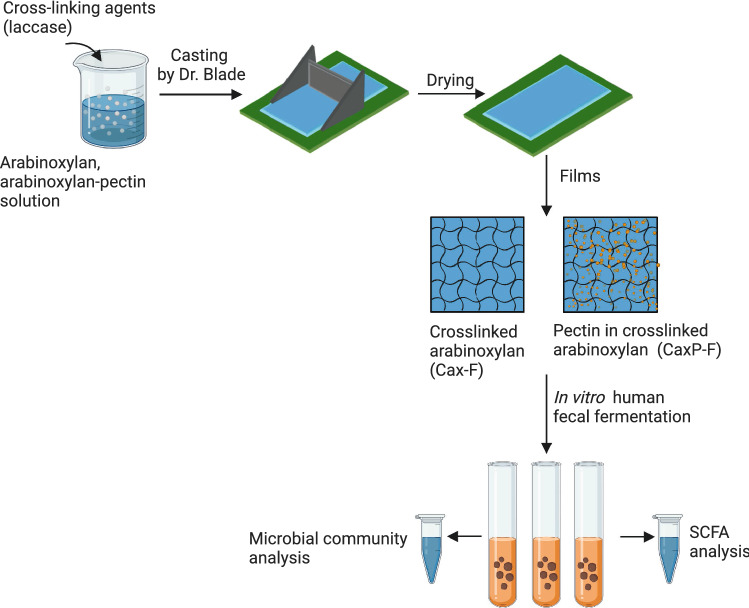
Diagram of film preparation and experimental design. The graphic summary depicts the film casting, the structure of the thin films, and the in vitro human fecal fermentation steps.

In this study, using this technique we fabricated cell wall-like crosslinked arabinoxylan thin films with and without embedded pectin to understand the effect of different cell wall fiber constituents and their physical form on the human gut microbiota and the fermentation SCFA metabolites (Fig. 1). We hypothesized when soluble arabinoxylan becomes crosslinked into an insoluble matrix it supports butyrogenic Clostridia bacteria and, further, that incorporation of pectin, as a soluble cell wall polysaccharide, into the insoluble matrix provides a narrower competitive niche for specific butyrogenic bacteria. Soluble pectin is a highly fermentable dietary fiber and has been shown to promote beneficial bacteria in the colon^22–24^, but inside the cell wall may target bacteria that can also degrade the crosslinked arabinoxylan. Hence, in vitro fecal fermentation profiles of these films (crosslinked arabinoxylan film [Cax-F], and crosslinked arabinoxylan and pectin film [CaxP-F]) were compared to their soluble forms (Cax and P). An improved understanding of how dietary fibers align, and support health-related gut bacteria is important to design and formulation of fibers in the diet for good gut health.

## Results

### Retention of fibers in the plant cell wall-like thin films

**Figure 2:**
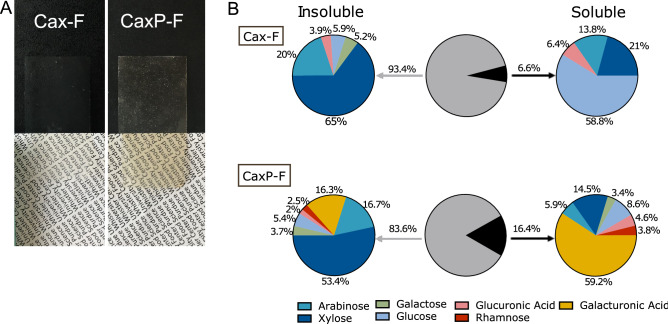
(**A**) Visual appearance of arabinoxylan film (Cax-F) and arabinoxylan film with embedded pectin (CaxP-F), (**B**) Monosaccharide profiles of insoluble and soluble portions of films. The pie chart in the middle represents the percentage of the films that was insoluble and soluble.

Crosslinked arabinoxylan formed insoluble thin films (Cax-F, Fig. 2A); solubility analysis showed that only about 7% was soluble when rehydrated at neutral pH. Also, the majority of the soluble nanosized pectin was entrapped inside the film when rehydrated (Fig. 2B). The films had a thickness of 15–17 μm for Cax-F and 18–20 μm for CaxP-F, which is in the range of an actual plant cell wall (10–20 μm)^25^. The insoluble Cax-F had a fairly typical corn bran arabinoxylan composition with a high xylose content (65%) followed by arabinose (20%), galactose (5%), glucuronic acid (4%) (6% glucose would be from residual starch). Insoluble CaxP-F was similar in proportional composition with the addition of ~ 19% of total composition as pectin monosaccharides (galacturonic acid and rhamnose). The minor ~ 7% Cax-F soluble fraction was mainly composed of glucose (~ 60%), likely caused by residual starch from the corn bran that was extracted with alkali. Xylose and arabinose, from the arabinoxylan, summed to 35% of the soluble fraction (Fig. 2B). In the CaxP-F, a larger amount of the film was soluble (16%), which was comprised mostly of pectin (galacturonic acid and rhamnose were 63% of the soluble carbohydrate). The smaller percentage of soluble carbohydrate in CaxP-F was arabinoxylan and residual starch (xylose, arabinose, and glucose summed to 36% of the film) (Fig. 2B). About 60% of incorporated pectin was retained in the insoluble film. Thus, the insoluble arabinoxylan-based film had sufficient intermolecular crosslinking to entrap a majority of the soluble pectin polymers in the matrix network.

### Short-chain fatty acid production

**Figure 3:**
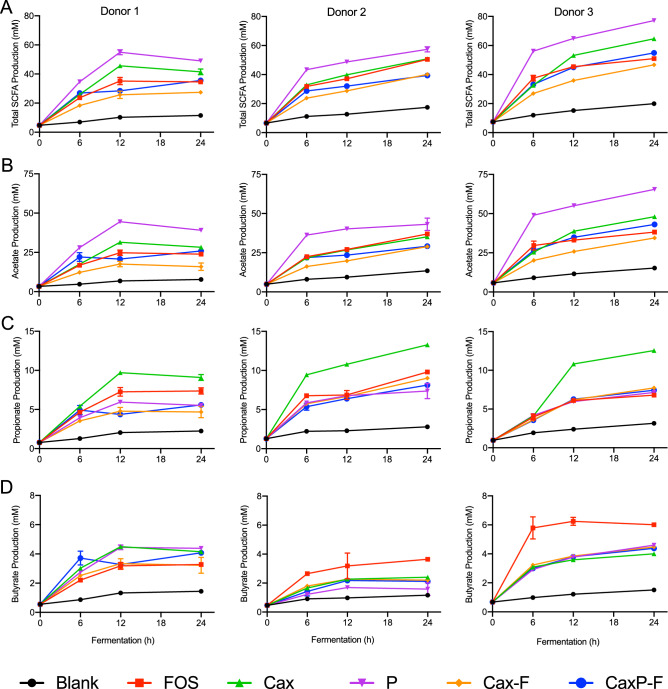
Short chain fatty acid (SCFA) production of 24 h in vitro fecal fermentations of soluble and film fibers in three donors; (**A**) total SCFA, (**B**) acetate, (**C**) propionate, and (**D**) butyrate. Fructooligosaccharide was the positive control. The blank was a negative control with no substrate. Error bars represent the standard error of the mean of three replicates. *FOS* fructooligosaccharides, *Cax* arabinoxylan, *P* pectin, *Cax*-*F* arabinoxylan film, *CaxP*-*F* arabinoxylan-pectin film.

SCFA production, as an indication of overall fermentability, decreased when the soluble Cax was crosslinked into the insoluble matrix film (Cax-F) (Fig. 3A). This was true in all three fecal donors. For each donor, free pectin was the most rapidly fermented substrate and soluble Cax and the fructooligosaccharide (FOS) control were the next most fermentable substrates. The crosslinked Cax film reduced fermentation compared to soluble Cax by about 25–40% at 24 h, depending on the donor. Entrapped pectin, found within the cell wall-like arabinoxylan matrices (CaxP-F) made the film more fermentable than the arabinoxylan film alone (28.5 and 19.5% higher total SCFAs at 24 h for Donors 1 and 3, respectively) (Fig. 3A).

Regarding individual SCFAs, pectin (a known acetogenic substrate^26,27^) resulted in the highest acetate levels in all three donors, and was followed by the group of Cax, FOS, and CaxP-F (Fig. 3B, Table S1). Note that pectin content in the CaxP-F was only one-quarter that of free pectin. Adding pectin in the form of the CaxP-F produced ~ 60 and ~ 25% more acetate compared to Cax-F for Donors 1 and 3, respectively (*P* < 0.05) (Fig. 3B, Fig. S1A). Cax generated significantly more acetate than Cax-F at 12 and 24 h for all donors (Fig. 3B, Table S1). Soluble Cax was highly propiogenic with the highest level of propionate generation at 12 to 24 h time points in all donors (Fig. 3C), supporting other studies showing that arabinoxylans are propiogenic^17,28,29^. Crosslinking of Cax into the insoluble film limited its utilization by propionate producers, as shown by the 30% to 45% reduction in propionate production of Cax-F compared to Cax, depending on the donor (Figs. 3C, S1B). Adding the pectin in the film (CaxP-F) did not change the level propionate compared to Cax-F in all donors (Fig. S1B).

Generally, there were no notable differences in total butyrate amounts among treatment groups, with the exception of FOS which showed significantly higher butyrate in Donors 2 and 3 compared to other substrates (*P* < 0.05) (Fig. 3D). Donor 1 produced more butyrate with fermentation of P and Cax. Fermentation of Cax, Cax-F, and CaxP-F at 24 h produced statistically the same amount of butyrate in Donors 1 and 2 (Table S1).

**Figure 4:**
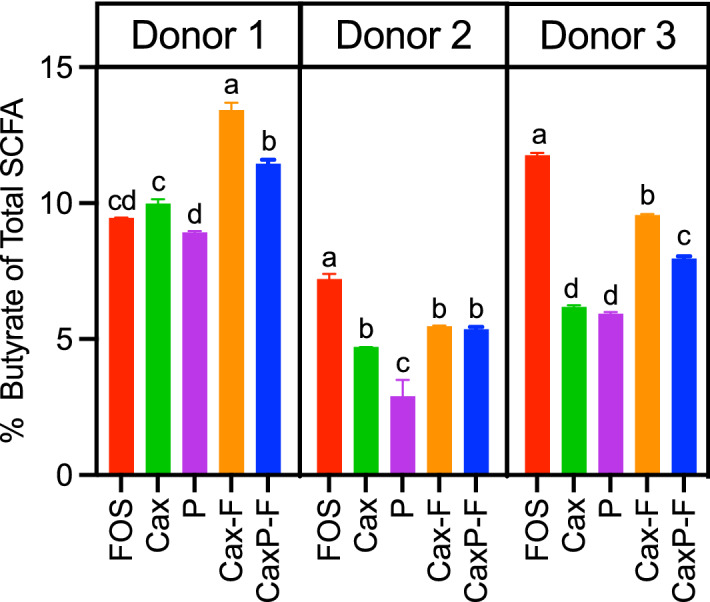
Butyrate percentage ratio generated at 24 h in vitro fecal fermentation in three donors. Error bars represent the standard error of the mean of three replicates. Different letters indicate significant differences in butyrate among treatments at the same time point (Tukey’s multiple comparison test, α = 0.05).

In Fig. 4, butyrate amounts per treatment are shown on a percentage basis of total SCFAs, which gives the view of whether butyrogenic bacteria were preferentially promoted by the fiber substrates. Butyrate percentages were significantly higher for the arabinoxylan-based films (Cax-F and CaxP-F) in Donors 1 and 3 than for their soluble counterparts (*P* < 0.0005).

### Microbiota shifts

**Figure 5:**
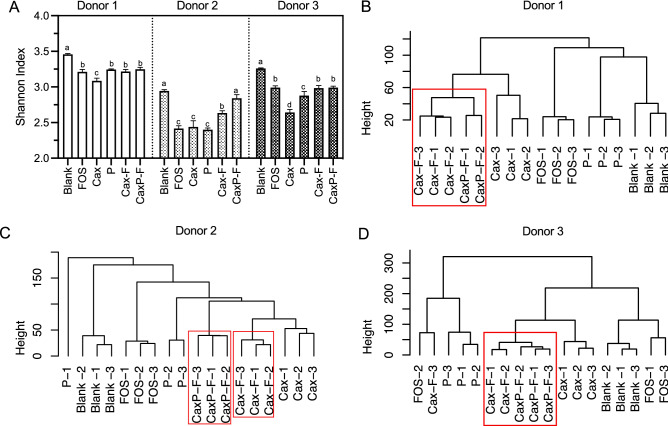
Microbiota diversity change after 24 h in vitro fecal fermentation; (**A**) Changes in Shannon alpha diversity for each donor fecal microbiota community of soluble and film fibers. Different letters indicate significant differences between substrates at the same time point (α = 0.05). Hierarchical cluster analysis tested fiber using the Ward agglomerative algorithm on Euclidean distances for (**B**) Donor 1, (**C**) Donor 2 and (**D**) Donor 3.

Alpha and beta diversities were measured to compare ferments of the cell wall-like films (Cax-F and CaxP-F), their original soluble forms (Cax, and P), the positive control (FOS), and the negative control (blank). Donor results were treated separately as there were differences found in community response to the different fibers. Alpha diversity was described by the Shannon diversity index which combines both richness and evenness of the microbial community (Fig. 5A). Most notably, alpha diversity was higher in all donors for the Cax film forms (Cax-F, CaxP-F) than for soluble Cax (*P* < 0.05), suggesting that an insoluble physical arrangement supports higher diversity. Moreover, in Donor 2, embedded pectin inside the arabinoxylan film (CaxP-F) promoted bacterial alpha diversity further, compared to Cax-F and other fiber ferments. Bray–Curtis beta diversity scores for 24 h ferments were not significantly different among treatments (PERMANOVA, *P* < 0.001) (Fig. S2).

Ward’s agglomerative algorithm on Euclidean distances was used to form a hierarchical clustering of the treatments in each donor (Fig. 5B–D). Cluster analysis shows a clear separation between Cax and the Cax films (Fig. 5B–D) confirming that distinct bacterial groups are promoted when Cax is in the insoluble film matrix form. In Donors 1 and 3 (Fig. 5B,D, respectively), Cax-F and CaxP-F were more similar to each other than Cax. In Donor 2 (Fig. 5C), Cax-F and Cax were more similar each other than CaxP-F.

**Figure 6:**
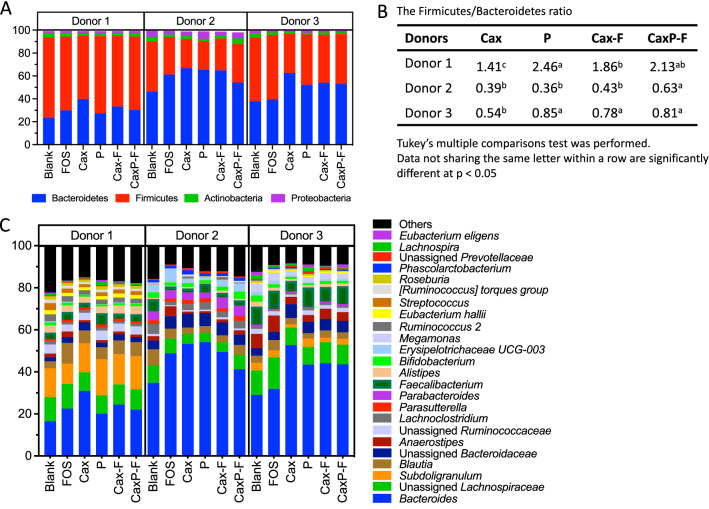
Microbiota composition change after 24 h in vitro fecal fermentation; (**A**) Phyla level (relative abundance > 1%) (**B**) Firmicutes/Bacteroidetes ratio Cax, P, Cax-F and CaxP-F in each donor (different letters represent significant differences [P < 0.05] between treatments), (**C**) Genus level (relative abundance > 0.5%).

Phylum and genus level changes within donor communities are shown in Fig. 6. Sequences derived from Firmicutes were dominant for Donor 1, while Bacteroidetes was the most abundant bacterial phylum in Donors 2 and 3 (Fig. 6A). In Donors 1 and 3, relative abundance of Firmicutes significantly increased after fermentation of the Cax film compared to soluble Cax (10% increase, Donor 1; 23%, Donor 3, *P* < 0.05). Addition of pectin to the film (CaxP-F) further promoted Firmicutes compared to the Cax film alone in Donor 2 (*P* < 0.05), and also trended higher in the other two donors. Firmicutes/Bacteroidetes ratio was also calculated (Fig. 6B). A significant increase in the Firmicutes/Bacteroidetes ratio was observed in Cax-F and CaxP-F fermentation when compared to Cax in Donors 1 and 3 (*P* < 0.05). Moreover, hiding pectin in arabinoxylan matrices promoted a significant increase in Firmicutes/Bacteroidetes ratio in Donor 2 compared to Cax-F, Cax and P (*P* < 0.05).

**Figure 7:**
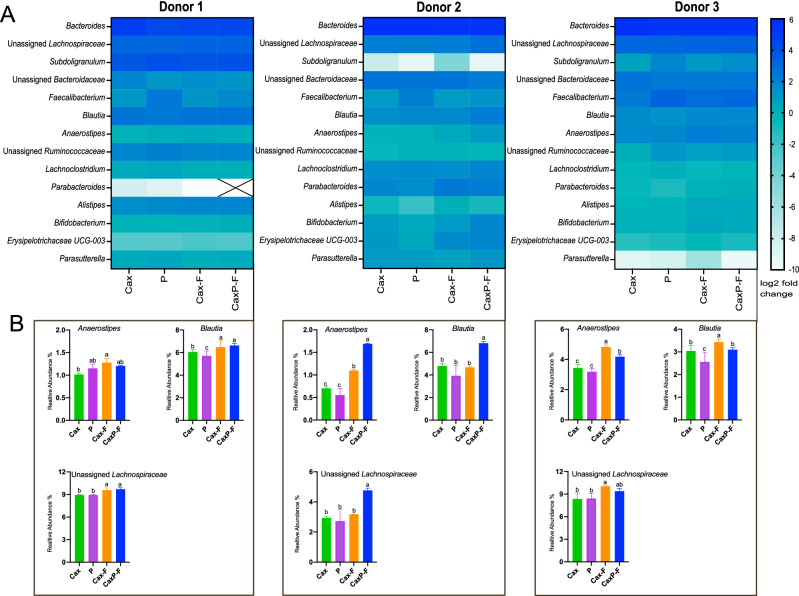
(**A**) Heatmap of the log_2_-transformed fold change at the genus level (relative abundance > 1%) after 24 h in vitro human fecal fermentation of Cax, P, Cax-F and CaxP-F for each donor. Each column represents the mean value of three replicates for each sample. (**B**) Relative abundances (%) of *Anaerostipes*, *Blautia* and unassigned *Lachnospiraceae* after 24 h in vitro human fecal fermentation for each donor. Error bars represent the standard error of the mean of three replicates. Different letters indicate significant differences among the treatments (Tukey’s multiple comparison test, α = 0.05).

**Figure 8:**
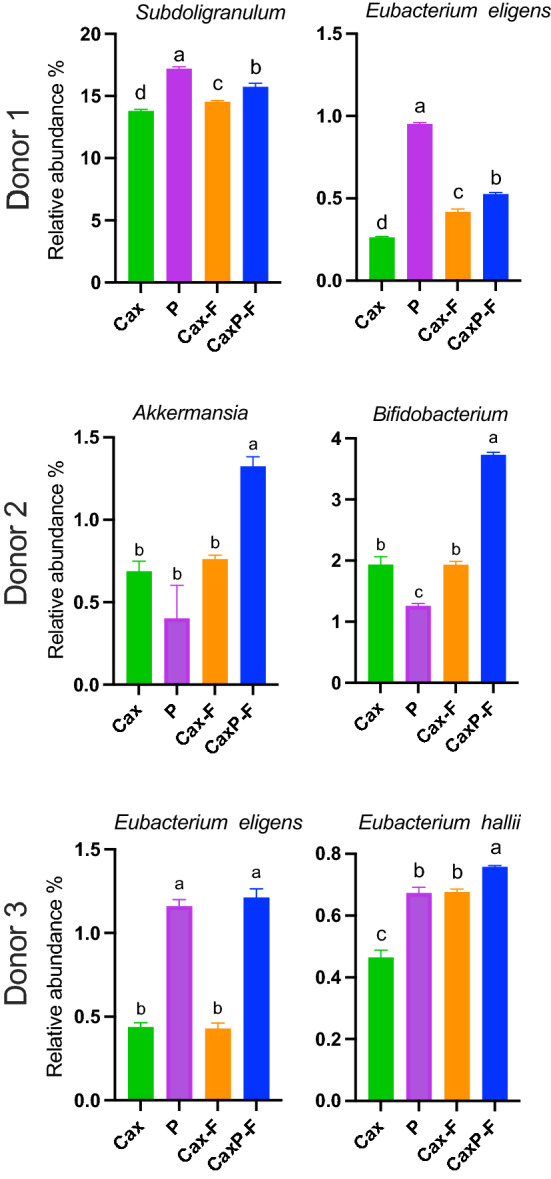
Select bacteria in each donor showing increase in relative abundance (%) in Cax film with embedded pectin. Error bars represent the standard error of the mean of three replicates. Different letters indicate significant differences among the substrates (Tukey’s multiple comparison test, α = 0.05).

At the genus level (relative abundance > 0.5%) (Fig. 6C), *Bacteroides* (phylum Bacteroidetes) was the most abundant genera for all three Donors, indicating that they are of the *Bacteroides* enterotype (Fig. 6C)^30^. In contrast, the phylum Firmicutes presented different taxa abundance across donors at the genus level (Fig. 6C). After fermentation with the treatment substrates, shifts in certain microbial taxa were observed with marked differences between insoluble Cax-F and CaxP-F versus their soluble counterparts (Fig. 7A). Either one or both matrices favored the growth of selected members of butyrogenic *Clostridium* cluster XIVa bacteria, such as *Anaerostipes*, *Blautia* and unassigned *Lachnospiraceae* in all donors (Fig. 7B). It was also notable in Donor 2 that *Anaerostipes, Blautia,* and unassigned *Lachnospiraceae* were significantly higher in relative abundance when in the entrapped pectin in crosslinked arabinoxylan film (CaxP-F), than for soluble pectin (P), native arabinoxylan (Cax), or the arabinoxylan film (Cax-F) (*P* < 0.05). CaxP-F increased the relative abundance of *Anaerostipes* 2.2 times more than Cax and 0.5 times more than Cax-F (Fig. 7). CaxP-F also preferentially promoted other bacterial taxa over Cax and Cax-F, such as *Subdoligranulum* (in Donor 1), *Eubacterium eligens* (in Donors 1 and 3), *Akkermansia* and *Bifidobacterium* (Donor 2) and *Eubacterium hallii* (Fig. 8).

## Discussion

In this study, two hypotheses were tested: (1) arabinoxylan existing in an insoluble cell wall-like film drives a different gut microbiota response (i.e. supporting Firmicutes) than if the same arabinoxylan is soluble, and (2) by placing a soluble fiber component of the cell wall (i.e. pectin) inside the cell wall-like film, it supports a narrow group of bacteria, including health-related species, that have the ability to access the fiber. To do this, a technology was established to make an insoluble arabinoxylan thin film similar to a plant cell wall in thickness and ferulate-crosslinking, and as well to place soluble pectin within it. Then, these fabricated insoluble fiber matrices and soluble counterparts were tested in an in vitro human fecal fermentation system (Fig. 1).

Key to making the insoluble arabinoxylan cell wall-like thin film was the preparation of the arabinoxylan. Corn bran arabinoxylan was solubilized using an alkali concentration low enough to de-esterify the diferulate crosslinks in the maize bran cell walls, but to retain a good amount of monoferulate residues on the solubilized arabinoxylan^20^. The arabinoxylan was then used with laccase, in a series of experiments, to reform crosslinks and make an insoluble thin film during the casting process. Crosslinking of ferulate residues turned arabinoxylan into Cax-F, representing a complex physical structure like the plant cell wall. Placing soluble pectin inside the fabricated film matrix tested the idea that a more complex insoluble fiber structure would further select for bacteria either having the ability to digest the crosslinked arabinoxylan network to access the pectin or that work in a consortium with other bacteria to degrade the cell wall material to get to the pectin substrate. This substrate resource-driven relationship with bacteria aligns with the view of niches that exist in the gut between bacteria and dietary fiber structures^7^.

Hopkins et al.^28^ previously showed that, although a crosslinked arabinoxylan gel was fermented more slowly than soluble arabinoxylan by gut microbiota, it did not affect individual bacterial groups except enterobacteria. Here, in perhaps a more highly crosslinked film version to simulate a cell wall-like structure, fermentation was likewise slower and significantly lower (*P* < 0.05) at all time points compared to the soluble Cax, but a number of bacteria were shifted in their relative abundance. Propionate proportion was lower in Cax-F than Cax which was related to decreased access of the fiber to propionate producers, *Prevotella* and *Bacteroides spp* (Fig. S3)^31,32^. In two of the donors, percent butyrate (of total SCFAs) was significantly higher in the Cax-based films (*P* < 0.05) (Fig. S2) and was related to a significant increase (*P* < 0.05) in Firmicutes/Bacteriodes ratio with coinciding increases in some butyrate-producing Firmicutes, such as *Anaerostipes, Blautia,* and unassigned *Lachnospiraceae* from *Clostridium* cluster XIVa (Fig. 6B). In fact, *Anaerostipes* was higher in all arabinoxylan films than in the soluble arabinoxylan and this was consistent in all donors. The major end products produced by *Anaerostipes* are lactate and butyrate^33^. Certain Firmicutes, including *Anaerostipes spp*., convert acetate into butyrate^34^. A number of butyrate-producers bacteria belong to *Lachnospiraceae* familiy that include the *Clostridium* cluster XIVa bacteria^35^, such as *Blautia* that was also increased in the fermentation of the films.

These results corroborate previous observations that commensal butyrogenic Clostridial bacteria preferentially colonize on insoluble complex fiber structures^10,35–37^. But more than just insolubility of fiber, it seems that matrices are the selecting factor to support these bacteria. In a study of ours mentioned above, Zhang et al.^10^ showed higher butyrate proportion and increases in butyrogenic Clostridia in fermentations with soluble crosslinked arabinoxylan matrices. Also, butyrate proportion was higher in more crosslinked arabinoxylan with higher ferulic acid content. Thus, re-crosslinking arabinoxylans into matrices may be an effective way to target and promote some of the Clostridial butyrate-producers.

To study a higher level of cell wall complexity in gut bacterial targeting, pectin was placed inside the crosslinked arabinoxylan film, thus making it largely insoluble. The resulting CaxP-F produced significantly higher total amount of short chain fatty acids than Cax-F in Donors 1 and 3. Furthermore, results from Donor 2 showed that the presence of pectin embedded into the arabinoxylan film provided a competitive niche to some butyrogenic bacteria in Clostridium cluster XIVa, such as *Anaerostipes*, unassigned *Lachnospiraceae*, and *Blautia*, and more than doubled the abundance of some other health-related bacteria (e.g. *Akkermansia* and *Bifidobacterium*). In other donors, pectin entrapment was associated with better promotion of *Subdoligranulum* (related to metabolic health)^38,39^ and *E. eligens* and *E. hallii* (both from *Clostridium* cluster XIVa) compared to Cax and Cax-F. Thus, a higher level of physical complexity of the fibers narrowed the group of bacteria that could utilize it.

The observation that the entrapped pectin in the insoluble arabinoxylan film (CaxP-F) targeted a bacterial response not seen in the P, Cax, or Cax-F treatments demonstrates the importance of physical arrangement of fibers and their fermentation in the gut. In our modern era, insoluble fermentable dietary fibers are the kind of fibers that have been reduced in refined “Western” diets, and this is associated with a reduction of several health-related taxa such as those from the butyrogenic *Clostridium* cluster XIVa and IV and *Akkermansia munciniphila*, among others^40^. Flint et al.^8^ reported that nutritionally specialized bacteria are critical to degrade complex substrates such as plant cell walls. Efforts to replenish fiber in processed foods have mainly focused on adding soluble dietary fibers^41^, which are not well suited for bacteria that have evolved to utilize fibers within insoluble matrices (i.e. plant cell walls). Here, we have shown that matrices containing insoluble and soluble-entrapped fibers provide specific opportunities for promotion of important health-related bacteria.

In conclusion, cell wall-like thin films were fabricated using fermentable cell wall fibers to understand gut bacterial degradation related to their physical form and location. We showed that the films gave a competitive niche to health-related Clostridia bacteria, boosting their growth. The more complex the cell wall-like film became, from an insoluble crosslinked arabinoxylan film to one with pectin embedded inside it, the more targeted it was to the promotion of specific bacterial taxa that are considered beneficial (e.g. *Eubacterium eligens*, *Akkermansia*, *Bifidobacterium*). Lastly, the work suggests that fabricated fermentable fiber structures can be designed with targeted function in the gut microbiota for good gut health.

## Methods

### Materials

Dry milled corn bran was gifted from Agricor, Ltd. (Marion, IN, USA). A commercial sample of low methoxy pectin from the citrus peel (900-69-5) and fructooligosaccharide (FOS—F8052) were purchased from Sigma-Aldrich (St. Louis, MO, USA). Thermostable α-amylase, laccase from *Trametes versicolor*, and methanolic-HCl were obtained from Sigma-Aldrich. Hexane, sodium hydroxide pellet, ethanol, concentrated HCl, and Tri-Sil were purchased from Fisher Scientific (Thermo Fisher Scientific, Suwanee, GA, USA).

### Arabinoxylan extraction

Arabinoxylan was extracted using the methodology described by Kale et al.^20^ with some modifications. Corn bran was defatted with hexane (bran: hexane, 1:7 [w/v]) and stirred two times for 45 min. The slurry was filtered, and the residue dried in a hot air oven at 45 °C. One-hundred grams of the defatted bran was suspended in 900 ml of water and pH was adjusted to 7.0 using 1 M NaOH. The mixture was boiled with constant stirring for 5 min to gelatinize the starch and then cooled to 90 °C. Thermostable α-amylase (4 mL) was added, and the de-starching reaction was done for 1 h at 90–95 °C. The slurry was centrifuged at 10,000*g* for 20 min. The supernatant was discarded, and the residue was washed twice with purified water and dried in a hot air oven at 45 °C to collect de-starched bran. The bran (50 g) was suspended in 500 mL of 0.25 M sodium hydroxide solution. After stirring for 30 min at room temperature, the suspension pH was adjusted to 4–5 using 1 M HCl. The slurry was centrifuged at 10,000*g* for 10 min. The residue was discarded, the volume of supernatant was measured, and an additional four volumes of absolute ethanol were added to precipitate the arabinoxylan, which was dried in a hot air oven at 45 °C. The arabinoxylan was re-dissolved in water and freeze-dried.

### Nanosized pectin preparation

A nanosized pectin powder was prepared by mechanical milling. Pectin (5 g) was milled using a Thinky Mixer (ARE-310, Thinky Corp., Laguna Hills, CA, USA) with 100 g of yttria-stabilized zirconia (YSZ) spherical grinding balls (2 mm in diameter) at 2400 rpm for 1 h. Dynamic light scattering (DLS) was used to analyze particle size of pectin using an ALV-CGS3 light scattering goniometer (ALV, Langen, Germany) with an HeNe laser (wavelength = 632.8 nm) at a 90-degree scattering angle.

### Film preparation

Two percent (w/v) arabinoxylan and arabinoxylan-pectin solutions were prepared by mixing the pure polymers in purified water for 30 min at 2400 rpm in the Thinky Mixer. Laccase (1.675 nkat/mg AX)^20^ was added as the crosslinking agent for the two solutions.

The arabinoxylan and arabinoxylan-pectin solutions were cast to a film on a glass platform by using a motorized drawdown coater equipped with a commercial 7.62 cm wide casting blade (Fig. 1). The casted films were 17.8 cm in length and 7.6 cm width and 760 μm thick. The films were cut and sieved to a size range of 0.5–1 mm for in vitro human fecal fermentation.

### Solubility analysis and the monosaccharide composition of soluble and insoluble portions of films

The solubility of films was measured as previously described^42^. First, the sieved films were dried at 105 °C for 24 h and initial weights were measured. Then, 100 mg samples were dissolved in 10 ml water and filtered using Whatman filter paper No.1. The residues were dried at 105 °C for 24 h and final weights were measured. Water solubility percentage was calculated based on Eq. (1).

1 $$Solubility=\left(\frac{{DM}_{I}-{DM}_{F}}{{DM}_{I}}\right)\times 100\%.$$

Monosaccharide compositions were performed on the soluble and insoluble portions of the films. Following the procedure above, residues and freeze-dried filtrates were used to determine neutral and acidic monosaccharides. Trimethylsilylation and methanolysis were performed according to Doco et al^43^. Trimethylsilyl (TMS) derivatives were analyzed by using gas chromatography coupled with mass spectroscopy (models 7890A and 5975C inert MSD with a Triple-Axis detector, Agilent Technologies, Inc., Santa Clara, CA, USA) (GC/MS) on a DB-5 capillary column (Agilent Technologies, Inc.)^43^. The monosaccharide compositions of arabinoxylan and pectin consisted of arabinose, xylose, galactose, glucose, rhamnose, galacturonic acid, and glucuronic acid amounts.

### In vitro fecal fermentation

Polysaccharide-based thin films (Cax-F, CaxP-F) and their original free forms (Cax, P), as well as positive (FOS) and negative (no substrate-containing blank) controls, were analyzed by in vitro human fecal fermentation to test the effect of physical positioning of plant cell wall-based fibers in soluble, insoluble film, and film-entrapped forms on metabolite and microbiota outcomes. For microbiota analysis, all treatments including the blank were analyzed at 24 h fermentation.

In vitro fecal fermentation was performed according to a previous paper^29^. Substrates (30 mg) were weighed into test tubes for each time point (0, 6, 12, 24 h). All substrates for each time point were prepared in triplicate and transferred to an anaerobic chamber. Carbon-phosphate buffer was prepared and sterilized by autoclaving at 121 °C for 20 min. After autoclaving, oxygen was removed by bubbling carbon dioxide, and 0.25 g/L of cysteine hydrochloride was added. The buffer was directly transferred into the anaerobic chamber at least one night before using. Carbonate-phosphate buffer (4 ml) was added to each tube containing substrates.

At the day of the experiment, feces were collected from 3 healthy donors who had consumed their routine diets and not taken antibiotics for at least 3 months. The collected fecal samples were sealed in plastic tubes and kept on ice and placed into the anaerobic chamber immediately. The feces were combined with three times the volume of carbonate-phosphate buffer and filtered with 4 layers of cheese cloth. Filtered fecal slurry (1 ml) was inoculated with the substrate in each tube. The tubes were sealed with rubber stoppers and incubated at 37 °C in a water bath with gently shaking.

The tubes were removed from the water bath at the planned time points. They were then opened, and the slurries were collected for DNA extraction (1 ml) and SCFA analysis (1 ml). Samples were stored at − 80 °C until further analysis.

### Short chain fatty acid analysis

The samples from the − 80 °C freezer were defrosted at room temperature and prepared for SCFA analysis as previously described^29^. The internal standard mixture for SCFA analysis was prepared using 4-methylvaleric acid, 85% phosphoric acid, and copper sulfate pentahydrate. Acetate, propionate, and butyrate were used as external standards. Samples were analyzed using a gas chromatograph (GC-FID 7890A, Agilent Technologies, Inc.) equipped with a fused silica capillary column (NukonTM, Supelco No: 40369-03A, Bellefonte, PA, USA) under the following conditions: injector temperature, 230 °C; detector temperature 230 °C; initial oven temperature, 100 °C; temperature program, 8 °C /min to 200 °C with a hold for 3 min at final temperature; carrier gas, helium at 0.75 ml/min. Quantification of short chain fatty acids was calculated from the peak areas of the acids relative to the internal standard. Total SCFA was measured as the sum of acetate, propionate, and butyrate, and relative proportions of each were determine.

### Microbiota analysis

Frozen fecal samples at 0 and 24 h were thawed and centrifuged at 13,000 rpm for 10 min for DNA extraction. The supernatants were discarded, and the pellets were homogenized in phosphate buffer. Then, the samples were transferred into lysing matrix E tubes from FastDNA SPIN® kit for feces (PC: 116,570,200) (MP Biomedical, Santa Ana, CA, USA). DNA was extracted according to the manufacturer’s instructions. The concentration of extracted DNA was evaluated using a Nanodrop ND-1000 spectrophotometer (Nanodrop Technology, Thermo Fisher Scientific).

Sequencing was performed using the Illumina MiSeq platform at the DNA Services Facility at the University of Illinois, Chicago. DNA was amplified using with PCR strategy with primers 515F and 806R. The V4 variable region of bacterial and archaeal small subunit (SSU) ribosomal RNA (rRNA) gene was amplified for each sample. Sequencing was performed on an Illumina MiSeq system.

### Bioinformatics

Sequences were analyzed using the quantitative insights into microbial ecology (QIIME2) version 2020.2 pipeline^44^. After demultiplexing and quality checking the FASTQ files, operational taxonomic units (OTU) were analyzed based on Greengenes database (version 13_8)^45^ identified with 97% similarity. Alpha diversity (Shannon) and beta diversity (PCoA; based on the Bray–Curtis methods) were calculated using the qiime2. The Bray–Curtis was statistically tested using PERMANOVA in R Stats Software version 3.5.1(R Core Team, Vienna, Austria). Other statistical analysis was performed in qiime2 and the graphs were generated and visualized using GraphPad Prism Software version 7.0 (GraphPad Software, Inc., La Jolla, CA, USA). Agglomerative hierarchical clustering analysis by Ward’s method on Euclidean distance was performed to cluster the dissimilarity among the treatments using Vegan package in R. To determine the statistical significance of the relative abundance of specific bacteria within treatments, one-way ANOVA was applied Tukey’s test for multiple comparison by using Minitab statistical software (release 16.0). Different letters represent the significant differences (P < 0.05) between treatments.

## Supplementary Information

Supplementary Information.

## Data Availability

Raw sequencing data were deposited in the National Center for Biotechnology Information Sequence Read Archive (NCBI; SRA) Bioproject PRJNA886103, and Biosamples SAMN31120033-095.
