# Supplementary material for: Matrix-entrapped fibers create ecological niches for gut bacterial growth
Source: Sci Rep. 2023 Feb 2;13:1884. doi: 10.1038/s41598-023-27907-7 (PMC9895076; doi:10.1038/s41598-023-27907-7)
Supplement: Supplementary file 1 — Supplementary Information. [file 41598_2023_27907_MOESM1_ESM.pdf]

Matrix-entrapped fibers create ecological niches for gut bacterial growth

Nuseybe Bulut[1], Thaisa M. Cantu-Jungles[1]*, Xiaowei Zhang[2], Zeynep Mutlu[3], Mukerrem Cakmak[3], Bruce R. Hamaker[1]*

Table S1. Statistical comparison of short-chain fatty acid (SCFA) production.

		Donor 1			Donor 2			Donor 3		
	Treatment/Time	6 h	12 h	24 h	6 h	12 h	24 h	6 h	12 h	24 h
**Total SCFA**	Blank	e	e	e	e	e	d	d	e	f
	FOS	c	c	c	bc	b	b	b	c	d
	Cax	bc	b	b	b	b	b	b	b	b
	P	a	a	a	a	a	a	a	a	a
	Cax-F	d	d	d	d	d	c	c	d	e
	CaxP-F	b	cd	c	c	c	c	b	c	c
**Acetate**	Blank	d	e	d	d	e	d	d	e	f
	FOS	bc	c	b	b	b	ab	b	c	d
	Cax	bc	b	b	bc	b	b	bc	b	b
	P	a	a	a	a	a	a	a	a	a
	Cax-F	c	d	c	c	d	c	c	d	e
	CaxP-F	b	cd	b	b	c	bc	b	c	c
**Propionate**	Blank	d	e	d	d	c	d	b	c	e
	FOS	abc	b	ab	b	b	b	a	b	d
	Cax	a	a	a	a	a	a	a	a	a
	P	bc	bc	c	bc	b	c	a	b	cd
	Cax-F	c	cd	c	bc	b	bc	a	b	b
	CaxP-F	ab	d	bc	c	b	bc	a	b	bc
**Butyrate**	Blank	c	c	c	c	c	c	c	c	d
	FOS	b	b	ab	a	a	a	a	a	a
	Cax	ab	a	ab	b	ab	b	b	b	c
	P	b	a	a	bc	ab	bc	b	b	b
	Cax-F	b	b	b	b	ab	b	b	b	b
	CaxP-F	a	b	ab	bc	ab	b	b	b	b

Different letters in the same column within the same SCFA category show significant differences (Tukey's multiple comparison test, $P < 0.05$).

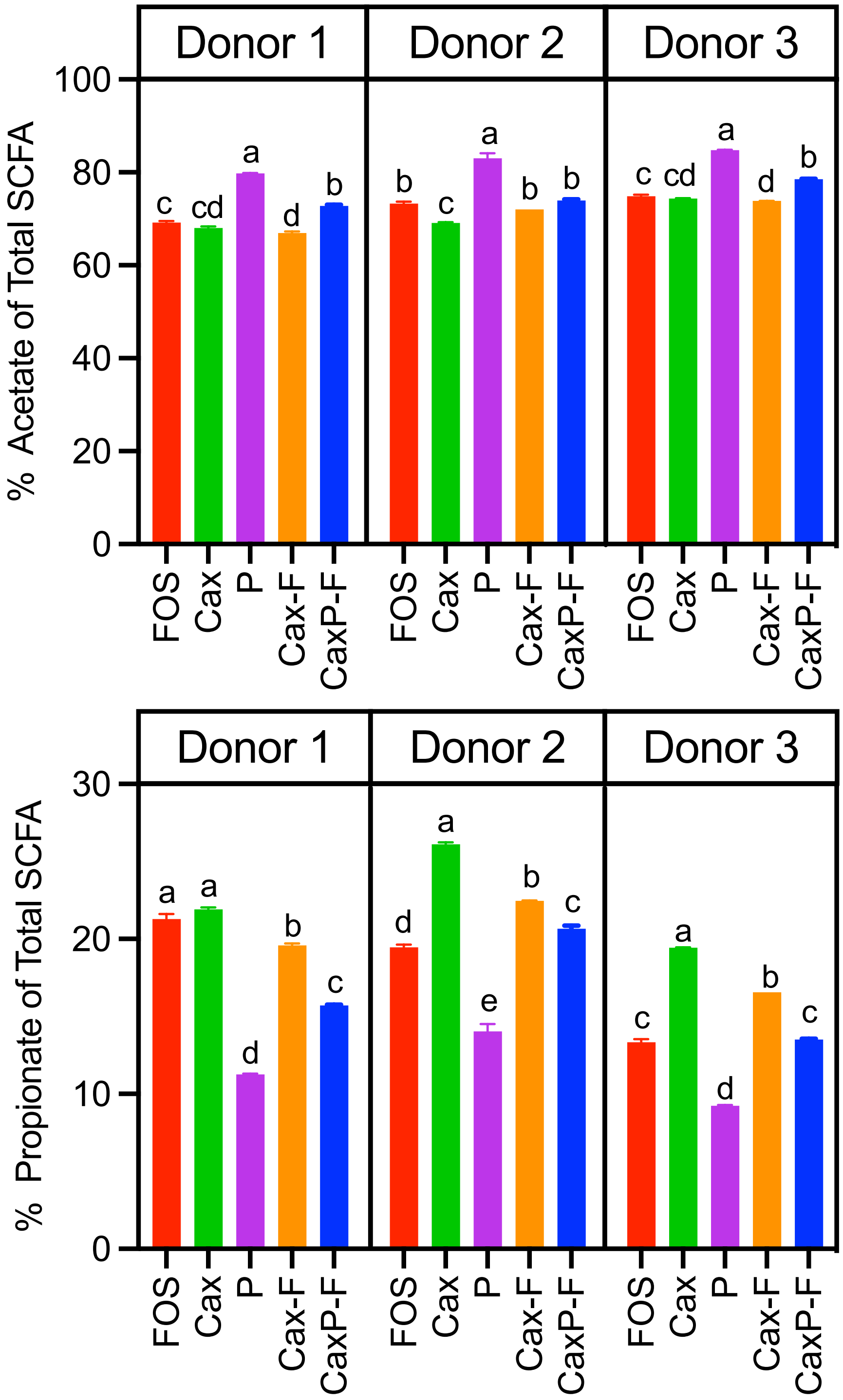

Figure S1. Acetate and propionate percentage ratio for all donors.

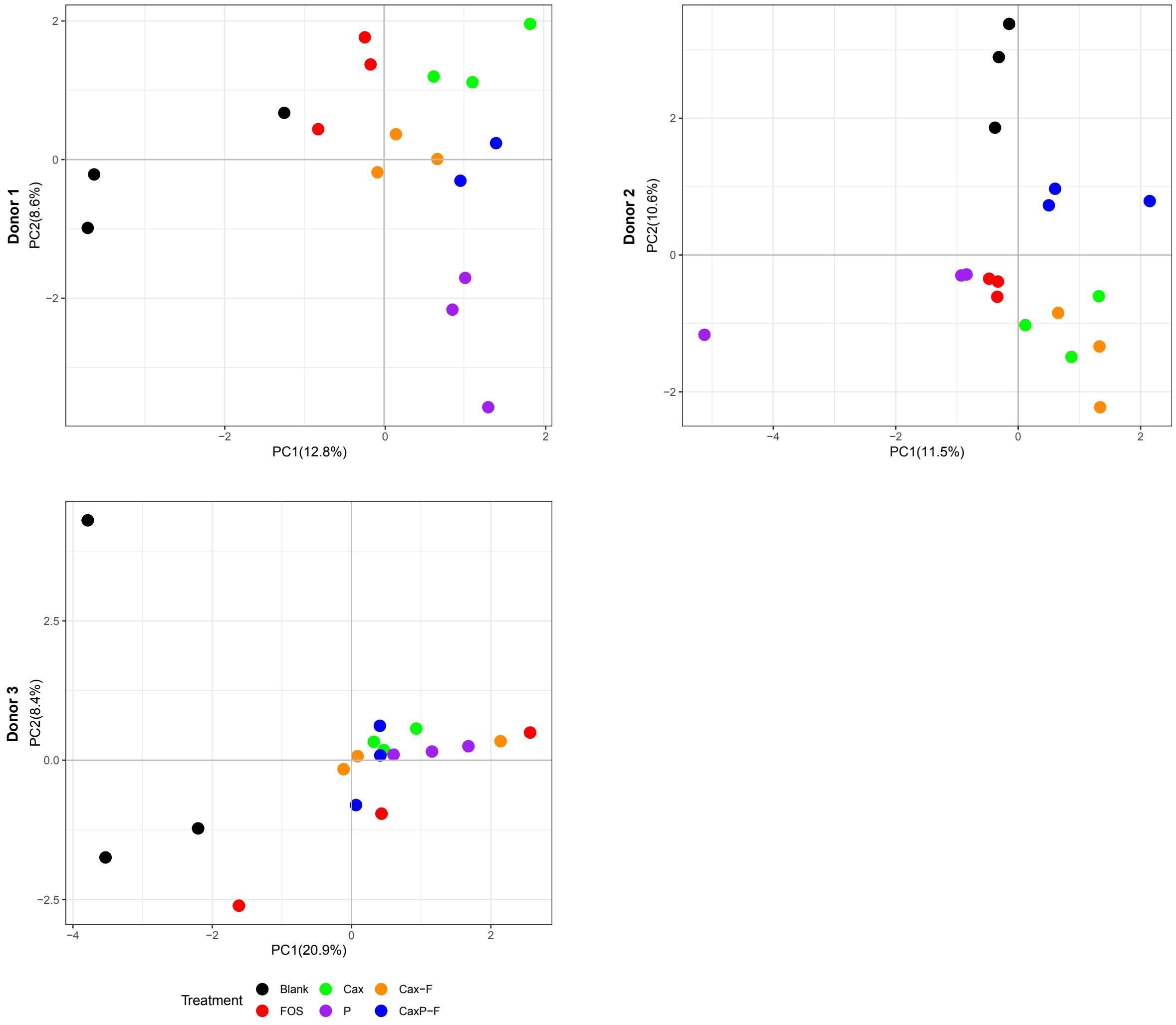

Figure S2. Principal component analysis of community structures of comparison of substrates based on donor 1, donor 2 and donor 3 as determined by 16S rRNA gene amplicon sequencing. Bray-Curtis dissimilarity of fecal microbiota was based on the relative abundances of OTUs at a 97% identity level after in vitro fermentation.

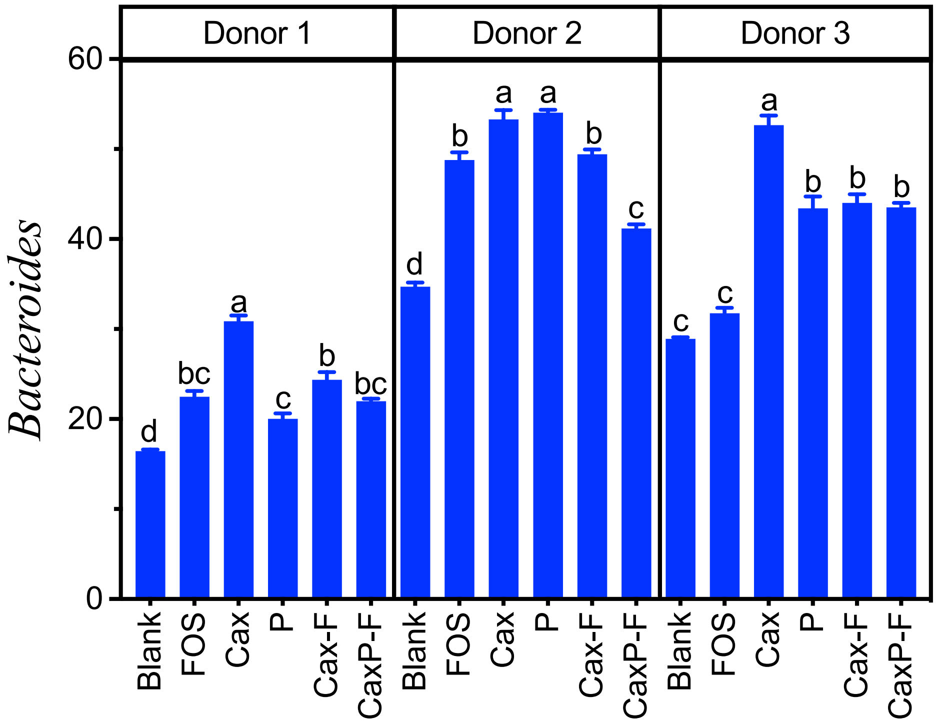

Figure S3. Relative abundances (%) of *Bacteroides* after 24 h *in vitro* human fecal fermentation for each donor. Error bars represent the standard error of the mean of three replicates. Different letters indicate significant differences among the treatments (Tukey's multiple comparison test, $\alpha = 0.05$).
